# Sexual Risk Behaviors and Current Substance Use Among Sexually Active Adolescents in the United States: Differences by Sex, Race, and Sexual Identity

**DOI:** 10.1007/s10935-026-00898-7

**Published:** 2026-01-28

**Authors:** Precious Patrick Edet, Hannah K. Allen, Ruaa Al Juboori, Azad R. Bhuiyan, Andrew Yockey

**Affiliations:** 1 William Magee Institute for Student Wellbeing, University of Mississippi, University, MS 38677 USA; 2 Department of Public Health, University of Mississippi, University, MS 38677 USA; 3 The Institute for Child Nutrition, University of Mississippi, University, MS 38677 USA; 4 Department of Epidemiology and Biostatistics, Jackson State University, Jackson, MS 39213 USA; 5 School of Nursing, University of Mississippi Medical Center, Jackson, MS 39216 USA

**Keywords:** Substance use, Sexual behavior, Prevention, Adolescents, YRBS, United States

## Abstract

Substance use and high-risk sexual behaviors remain pressing public health challenges among U.S. adolescents, with tobacco, alcohol, and marijuana being the most used substances. While previous research has shown an association between substance use and risky sexual behavior, there is limited data on how these associations vary based on demographic modifiers such as race, sex, and sexual identity among sexually active adolescents. This study explores the association between current substance use (cigarettes, e-cigarettes, alcohol, and marijuana) and high-risk sexual behaviors (having multiple sexual partners and lack of condom use), examining how these associations vary by race, sex, and sexual identity. The 2023 Youth Risk Behavior Survey (YRBS) data was analyzed for 5420 adolescents attending U.S. public and private high schools. Multiple Logistic regression was used to examine associations, and interaction effects for race, sex, and sexual identity were introduced to the model to determine variations in associations. Overall adjusted analyses revealed current use of cigarette, e-cigarette, and alcohol was associated with higher likelihood of having multiple (2 or more) sexual partners in the past 3 months. Additionally, current marijuana use was associated with a higher likelihood of not using a condom during last sexual intercourse. A statistically significant interaction was observed only for the association between current marijuana use and condomless sex during last sexual intercourse (*p* = 0.010). Stratified analyses indicated that this association varied by sexual identity and was stronger among heterosexual students compared to Lesbian, Gay, Bisexual, Queer, and other (LGBQ+) students. Results confirm the link between substance use and high-risk sexual behavior among youth, reinforcing our need for increased programming around substance use prevention and sexual health education. Heterosexual youth may be at increased risk for high-risk sexual behavior associated with their marijuana use, calling for tailored interventions that target the unique needs of this demographic subgroup.

## Introduction

Substance use and high-risk sexual behaviors among adolescents remain critical public health concerns in the United States (U. S.), given their implications for health outcomes such as sexually transmitted infections (STIs), including HIV, unintended pregnancies, psychiatric disorders in adulthood, to name a few (Centers for Disease Control & Prevention, 2020; Hops et al., 2011; Steinfeld & Torregrossa, 2023). Adolescence, characterized by experimentation and identity formation, is a period during which many risky behaviors, including substance use and sexual risk-taking, emerge (Ruprah et al., 2017).

Tobacco, alcohol, and marijuana are the most common substances used by adolescents (Bagley, 2024; Edet & Mendy, 2023; Khurana et al., 2015; National Institute on Drug Abuse [NIDA], 2022). In 2024, approximately 2.25 million middle and high school students reported using any tobacco product at least once in the past 30 days (U.S. Food & Drug Administration [FDA], 2025). The 2023 National Survey on Drug Use and Health (NSDUH) found that 12.6 million individuals aged 12 to 20, representing 33.1% of this age group, reported consuming alcohol at least once in their lifetime (National Institute of Alcohol Abuse & Alcoholism [NIAAA], 2024). Over the past decade, the percentage of adolescents who view weekly cannabis use as harmful has declined significantly from 47.5% to 27.4%, while cannabis use among this population has increased from 11.6% to 17.9% (Sultan et al., 2023). Additionally, adolescents who use substances are more likely to report high-risk sexual behaviors, including early initiation of sexual activity, multiple sexual partners, inconsistent condom use, and sex under the influence of substances (Clark et al., 2020; Clayton et al., 2019; Ritchwood et al., 2015).

Research has highlighted disparities in both substance use and sexual risk behaviors among subgroups of adolescents. For instance, Goodwin and Silverman (2024) found that, in 2021, cannabis use was more prevalent among female than male adolescents, and among non-Hispanic Black and Hispanic adolescents compared to their non-Hispanic White peers. Additionally, sexual minority youth—those identifying as lesbian, gay, bisexual, transgender, or questioning (LGBTQ+)—experience disproportionately higher rates of both substance use and sexual risk behaviors compared to their heterosexual peers (Marshal et al., 2008; Rasberry et al., 2018). Factors such as minority stress, lack of social support, and stigma have been proposed as contributors to these disparities (Hatzenbuehler, 2009; Hatzenbuehler et al., 2015). Moreover, heightened stigma-related stress exacerbates emotional dysregulation and disrupts cognitive function, which in turn fosters maladaptive coping mechanisms, including substance use, thereby perpetuating a harmful cycle of psychological and behavioral dysfunction (Clayton et al., 2019).

Differences by race and sex in the association between high-risk sexual behavior and substance use have been found in prior research. Substance use, especially alcohol and marijuana, has been identified as a predictor of risky sexual behavior in both White and African American adolescents (Banks et al., 2016). However, alcohol use is more strongly associated with risky sexual behavior in White adolescents compared to African American adolescents, likely due to the considerably higher prevalence of alcohol use among White adolescents (Banks et al., 2016), though a recent report shows a higher prevalence among African American students in 12th grade compared to their White peers (Miech et al., 2023). Additionally, male adolescents who consume alcohol are 2.9 times more likely than their female peers to report having multiple sexual partners (Cho & Yang, 2023).

While the association between substance use and sexual risk behaviors has been documented over the years, less is known about how these associations vary by race, sex, and sexual identity among sexually active U.S. adolescents. Understanding these variations is essential for tailoring interventions to address the unique needs in subpopulations. Clayton et al. (2019) explored sexual identity variations in the association between substance use and risky sexual behaviors among U.S. adolescents using the 2015 Youth Risk Behavior Survey (YRBS) data. Their findings revealed that while most associations were consistent across sexual identities, certain substance use behaviors were more strongly associated with risky sexual behaviors among heterosexual students compared to their LGB counterparts. However, further research is needed to explore how this association varies among sexually active adolescents across different demographic groups using more recent data.

This study builds on previous research by examining variations in the association between current substance use and high-risk sexual behaviors among sexually active adolescents in the United States using the 2023 YRBS data. Specifically, this study aims to assess the overall association between current substance use and high-risk sexual behaviors among sexually active adolescents in the U.S., controlling for demographic factors such as age, race, sex, grade, and sexual identity. Additionally, this study will examine variations in this association by race, sex, and sexual identity. We hypothesize that current substance use will be significantly associated with high-risk sexual behavior, and variations in these associations will be present.

Implications of this study are central to the field of prevention because effective adolescent health interventions rely on understanding which populations are most vulnerable and why (Aagaard-Hansen et al., 2023). For example, sex- and race-specific differences in both substance use and sexual risk behaviors have been documented (Banks et al., 2016; Cho & Yang, 2023; Miech et al., 2023), highlighting the need for tailored prevention strategies. Recognizing these differences allows public health practitioners and policymakers to design culturally responsive and developmentally appropriate interventions, such as programs that integrate substance use education with sexual health promotion, to address disparities in health outcomes. By identifying demographic variations, this study provides evidence to inform targeted school- and community-based prevention programs, enhancing intervention effectiveness and equity in adolescent health.

## Methods

### Participants and Procedures

The YRBS is a nationwide, school-based survey conducted every two years since 1991 during the spring semester by the CDC (2024a). However, because of the COVID-19 pandemic, most state, territorial, tribal government, and local surveys were administered during the fall semester in 2023 (CDC, 2024a). The YRBS survey focuses on high school students in grades 9–12 from public and private schools across all 50 states and the District of Columbia (CDC, 2024a). The survey uses a three-stage cluster sampling method to ensure the data reflects the broader student population while keeping responses anonymous (CDC, 2024a). To make the data representative, weights are applied to account for factors like nonresponse and the oversampling of Black and Hispanic students (CDC, 2024a). Final weights are further adjusted to match weighted student totals with the overall sample size, while ensuring that grade-level proportions correspond to population projections for each survey year (CDC, 2024a). This description is consistent with methodologies used in previous YRBS-based studies (Clayton et al., 2019; Mittleman, 2025; Zhang et al., 2024).

In 2023, the YRBS collected data from 20,103 high school students, including a total of 5,420 sexually active high school students selected for this study specifically. The survey followed strict protocols on parental consent requirements (CDC, 2024a). The 2023 dataset is publicly available and approved by the CDC’s Institutional Review Board (CDC, 2024a).

### Measures

Four substance use behaviors were assessed for this study as exposure variables: (1) current cigarette smoking, (2) current e-cigarette use, (3) current alcohol use, and (4) current marijuana use. Participants were asked how often they used each substance in the past 30 days. Responses were dichotomized into “Yes” or “No”, with responses including 1 or more days or times coded as “Yes” and responses including “0 days” or “0 times” coded as “No.”

Two sexual risk behaviors were evaluated for this study as outcome variables, corresponding to indicators established by the CDC and other research studies. These included: (1) having 2 or more sexual partners within the past 3 months, and (2) not using a condom during last sexual intercourse. According to Rosenberg (2004), Santelli et al. (1998), and Ssebunya et al. (2019), having 2 or more sexual partners within the past 3 months is considered a high-risk sexual behavior. Additionally, lack of condom use during the last sexual intercourse is also considered a risky sexual behavior (Chawla & Sarkar, 2019; Clayton et al., 2019). To assess sexual risk behaviors, respondents were asked “During the past 3 months, with how many people did you have sexual intercourse?” Responses that indicated having sexual intercourse outside the 3-month window or had intercourse with only one person were not considered to be at sexual risk, so coded as “No.” Respondents who reported having 2 or more sexual partners within the past 3 months were considered to exhibit high-risk sexual behavior, so their response was coded as “Yes.” Additionally, respondents were asked “The last time you had sexual intercourse, did you or your partner use a condom?” Responses were coded as “Yes” or “No.”

Demographic variables served as covariates for this study and included race/ethnicity, age group, sex, grade level, and sexual identity. Race/ethnicity data comprised American Indian/Alaskan Native, Asian, Black or African American, White, Hispanic/Latino, and other (Multiple-Hispanic, Multiple-Non-Hispanic, Native Hawaiian/Other Pacific Islander) groups. Age group data comprised 13-year-olds or younger, 14–16-year-olds, and 17 or older groups. Respondents reported being either male or female and in either 9th, 10th, 11th, or 12th grade. Sexual identity data comprised heterosexual, lesbian, gay, bisexual, questioning, and other identity groups.

To assess variation in associations between current substance use and high-risk sexual behavior, the data was stratified by race, sex, and sexual identity. For ease of analysis, the race variable was dichotomized to “White” and “Non-White” categories, while sexual identity was dichotomized to “Heterosexual” and “LGBQ+” categories.

### Data Analysis

We conducted descriptive statistics to examine demographic characteristics, current substance use, and sexual risk behaviors within the study population. To identify significant variations in the relationships between current substance use and sexual risk behaviors, we performed multiple logistic regression for both overall and stratified models (stratified by race, sex, and sexual identity), adjusting for covariates. These analyses produced adjusted odds ratios (aORs) with 95% confidence intervals. Statistical significance was determined if the 95% confidence interval did not include 1. Prior to performing a logistic regression, multicollinearity was assessed and ruled out in the model, as all independent variables and covariates had a variance inflation factor (VIF) below 5.

To assess whether the associations between current substance use and high-risk sexual behaviors varied across key demographic characteristics, effect modification by race, sex, and sexual identity was evaluated in the overall logistic regression models. Interaction terms between each substance use variable (current cigarette use, e-cigarette use, alcohol use, and marijuana use) and race, sex, and sexual identity were added separately to models predicting (1) having multiple sexual partners in the past 3 months and (2) condomless sex during last sexual intercourse, while adjusting for age group, race, sex, grade level, and sexual identity as appropriate. Interactions with p-values less than 0.05 were considered statistically significant.

Covariates in the overall model included age group, race, sex, grade level, and sexual identity. These variables were controlled for to account for known demographic differences in both substance use and high-risk sexual behaviors among adolescents, as highlighted in prior research (Banks et al., 2016; Godwin & Silverman, 2024; Miech et al., 2023; Rasberry et al., 2018). Controlling for these covariates therefore helps reduce confounding in the models. This analytic approach is consistent with previous YRBS-based analyses, including Clayton et al. (2019), which adjusted for similar demographic covariates. Listwise deletion, the default approach in SAS, was used to handle missing data for analytic variables as less than 5% of the data was missing. Comprehensive details regarding the national YRBS sampling methods and the psychometric evaluation of the YRBS questionnaire can be found in prior publications (Brener et al., 2002, 2013; Clayton et al., 2019). Table 1 provides details on dependent and independent variables used in this study.

**Table 1:** Current substance use and high-risk sexual behaviors among sexually active U.S. adolescents, youth risk behavior survey

Adolescent behaviors	Questionnaire item	Analytic coding
Current cigarette use	During the past 30 days, on how many days did you smoke cigarettes?	Yes (≥ 1 day) versus No (0 days)
Current e-cigarette use	During the past 30 days, on how many days did you use an electronic vapor product?	Yes (≥ 1 day) versus No (0 days)
Current alcohol use	During the past 30 days, on how many days did you have at least one drink of alcohol?	Yes (≥ 1 day) versus No (0 days)
Current marijuana use	During the past 30 days, how many times did you use marijuana?	Yes (≥ 1 times) versus No (0 times)
Having multiple sexual partners in ≤ 3 months	During the past 3 months, with how many people did you have sexual intercourse?	Yes (≥ 2 persons) versus No (< 2 person)
No condom use during last sexual intercourse	The last time you had sexual intercourse, did you or your partner use a condom?	Yes versus No

The analysis was conducted using SAS version 9.4 (SAS Institute Inc., 2024). To account for the complex sampling design, the data was weighted, clustered, and stratified using the WEIGHT, PSU, and STRATUM variables, respectively, and proc surveyfreq and proc surveylogistic procedures.

## Results

### Demographic Characteristics

A total of 5,420 sexually active high school students were included in the analysis. Approximately 47% were female and 74% identified as heterosexual compared to their male and LGBQ + counterparts, respectively. Racial distribution showed that the majority of students were White (50%), followed by “other” (27%), Black/African American (14%), Hispanic (6%), Asian (2%), and American Indian/Native American (0.4%). The majority of students were 17 years or older (54%), followed by 14–16-year-olds (46%), and least among 13-year-olds or younger (0.4%). Additionally, the majority of students were in 12th grade (37%).

Findings on current substance use showed that 41.4% drank alcohol, 36.5% used e-cigarettes, 36.1% used marijuana, and 8% of students smoked cigarettes. Regarding sexual risk behaviors, 11.4% of students reported having sexual intercourse with two or more partners in the past 3 months, and 54.4% reported that either they or their partner used a condom during their last sexual encounter. Table 2 presents comprehensive demographic information for the study population.

**Table 2:** Characteristics of the study population of sexually active adolescents in the United States, youth risk behavior surveillance system, 2023

Characteristics	n^a^	N^b^	%^c^	[95% CI]
**Age group**				
≤ 13 years	22	22	0.4	[0.1–0.7]
14–16 years	2578	2469	45.6	[43.0–48.3]
≥ 17 years	2786	2919	54.0	[51.3–56.6]
**Race/ethnicity**				
American Indian	430	19	0.4	[0.3–0.5]
Asian	96	93	1.7	[0.9–2.6]
Black	568	772	14.4	[8.3–20.4]
White	2609	2690	50.1	[43.6–56.6]
Hispanic	194	323	6.0	[3.9–8.2]
Other	1467	1472	27.4	[23.5–31.3]
**Sex**				
Male	2774	2845	47.3	[42.3–50.3]
Female	2616	2557	52.7	[49.7–55.7]
**Sexual identity**				
Heterosexual	3613	3851	77.2	[74.8–79.6]
Lesbian or Gay	189	225	4.5	[3.7–5.4]
Bisexual	709	766	15.4	[13.5–17.2]
Questioning	166	146	2.9	[2.2–3.7]
Other (+)	180	189	3.7	[2.6–5.0]
**Grade level**				
9th	761	725	13.5	[11.6–15.4]
10th	1194	1092	20.4	[18.3–22.4]
11th	1586	1568	29.2	[25.8–32.7]
12th	1800	1979	36.9	[33.2–40.6]
**Current cigarette use**				
Yes	506	430	8.0	[6.3–9.7]
No	4846	4947	92.0	[90.3–93.7]
**Current E-cigarette use**				
Yes	1966	1858	36.5	[33.0–39.9]
No	3092	3235	63.5	[60.1–67.0]
**Current alcohol use**				
Yes	2128	2166	41.4	[37.9–44.8]
No	3101	3071	58.6	[55.2–62.1]
**Current marijuana use**				
Yes	1976	1924	36.1	[33.3–39.0]
No	3331	3400	63.9	[61.0–66.7]
**Multiple sexual partners in < 3 months**				
Yes	640	575	11.4	[9.7–13.1]
No	4524	4476	88.6	[86.9–90.3]
**Condom use**				
Yes	2733	2723	54.4	[51.6–57.3]
No	2414	2282	45.6	[42.7–48.4]

n^a^ unweighted number, N^b^ weighted number, %^c^ weighted percentage, CI confidence interval

### Multiple Logistic Regression Analysis

#### Overall Analysis

Logistic regression analysis of the overall sample revealed significant associations between current substance use and high-risk sexual behaviors after adjusting for age group, race, sex, grade level, and sexual identity. Specifically, students who reported current use of cigarettes, e-cigarettes, and alcohol were each 1.9 times more likely to have had two or more sexual partners in the past 3 months compared to their non-using peers (95% CI for cigarette: 1.2–3.1; e-cigarette: 1.2–3.3; alcohol: 1.3–2.8). Students who currently used marijuana were 1.6 (95% CI 1.2–2.1) times more likely to report not using a condom during their last sexual encounter compared to non-users. There were no associations between current marijuana use and having multiple sexual partners in the last 3 months, and between current use of cigarette, e-cigarette, and alcohol and no condom use during last sexual intercourse.

Table 3 outlines the associations between current substance use and risky sexual behavior across the overall sample.

**Table 3:** Multiple logistic regression analysis on the association between current substance use and high-risk sexual behavior among sexually active adolescents in the United States, youth risk behavior surveillance system, 2023

Current substance use behaviors	2 or more partners in ≤ 3 months aOR (95%CI)	*p*-value	% (95%CI)	No condom use during last sex aOR (95%CI)	*p*-value	% (95%CI)
Cigarette use	**1.9 (1.2–3.1)**	**0.007**	28.0 (22.2–33.8)	1.4 (0.8–2.5)	0.268	61.2 (51.4–71.1)
E-cigarette use	**1.9 (1.2–3.3)**	**0.012**	18.2 (15.3–21.0)	1.4 (1.0–1.9)	0.051	55.4 (51.7–59.0)
Alcohol use	**1.9 (1.3–2.8)**	**0.001**	16.5 (14.2–18.8)	0.9 (0.7–1.1)	0.280	49.2 (45.6–52.9)
Marijuana use	1.2 (0.9**–**1.6)	0.148	17.6 (14.4–20.7)	**1.6 (1.2–2.1)**	**0.013**	56.0 (51.6–60.4)

Reference group for each substance use variable: No current use

Adjusted covariates: race, sex, age-group, grade level, and sexual identity

%, unadjusted weighted prevalence of substance use behaviors

aOR adjusted odds ratio, CI confidence interval

Statistically significant results based on 95% CI and *p* < 0.05 are bolded

#### Effect Modification Analysis

Among all interaction terms tested, only sexual identity significantly modified the association between current marijuana use and condomless sex during last sexual intercourse. No significant effect modification by race or sex was observed for any substance use–sexual behavior associations. Based on this finding, stratified analyses were conducted to further examine the relationship between current marijuana use and condomless sex during last sexual intercourse by sexual identity (heterosexual vs. LGBQ+).

#### Stratification Analysis

Stratification by sexual identity revealed that the significant association between current marijuana use and condomless sex during last sexual intercourse observed in the overall sample remained significant only among heterosexual students (aOR: 1.8; 95% CI 1.1–2.8) but not among LGBQ + (aOR: 1.1; 95% CI 0.7–1.5) students, indicating a variation in this association, with a stronger association among heterosexual students compared to their LGBQ + counterparts.

Results of the effect modification analyses examining race, sex, and sexual identity as modifiers of the associations between current substance use and high-risk sexual behaviors are presented in Tables 4, 5.

**Table 4:** Interaction coefficients (β) and *p*-values for effect modification on the association between current substance use and having multiple sexual partners among sexually active adolescents in the United States, youth risk behavior surveillance system, 2023

Current substance use behavior	Race		Sex		Sexual identity	
	B coefficient	*p*-value	B coefficient	*p*-value	B coefficient	*p*-value
Cigarette	−0.129	0.785	0.271	0.505	0.053	0.911
E-cigarette	0.527	0.185	−0.338	0.466	−0.147	0.797
Alcohol	−0.434	0.214	−0.615	0.115	0.736	0.086
Marijuana	−0.198	0.527	0.207	0.647	−0.137	0.772

β coefficient: Interaction β coefficient

Reference group for each substance use variable: No current use

Statistically significant results based on *p* < 0.05 are bolded

**Table 5:** Interaction coefficients (β) and *p*-values for effect modification on the association between current substance use and condomless sex during last sexual intercourse among sexually active adolescents in the United States, youth risk behavior surveillance system, 2023

Current substance use behavior	Race		Sex		Sexual identity	
	B coefficient	*p*-value	B coefficient	*p*-value	B coefficient	*p*-value
Cigarette	0.449	0.350	0.234	0.713	0.059	0.911
E-cigarette	−0.155	0.563	−0.034	0.887	0.355	0.182
Alcohol	0.314	0.256	−0.272	0.345	0.409	0.105
Marijuana	0.363	0.087	−0.126	0.665	−0.640	**0.010**

β coefficient: Interaction β coefficient

Reference group for each substance use variable: No current use

Statistically significant results based on *p* < 0.05 are bolded

## Discussion

The findings of this study highlight critical insights into the relationship between current substance use and high-risk sexual behaviors among sexually active adolescents in the U.S., both overall and across stratified groups. Consistent with prior research (Chawla & Sarkar, 2019; Clayton et al., 2019; Ritchwood et al., 2015), current substance use, including cigarette, e-cigarette, alcohol, and marijuana, was significantly associated with high-risk sexual behaviors in the overall analysis, reaffirming the importance of addressing these behaviors within adolescent populations to reduce adverse health outcomes.

In the overall analysis, the results reveal that adolescents currently using cigarettes, e-cigarettes, and alcohol were significantly more likely to report having two or more sexual partners in the past 3 months. These findings are similar to findings from Guo et al. (2017) study conducted in China. Their study, which utilized a nationally representative data of youths aged 15–24, found that individuals who used cigarette and alcohol had a higher likelihood (OR: 4.9, 95% CI 3.8–6.2) of engaging in sexual activity with multiple partners than their non-using peers (Guo et al., 2017). Additionally, a study by Chang and Seo (2021), which utilized the 2017 YRBS data for 14,965 U.S. high school students, revealed an association between current e-cigarette use and having multiple sexual partners (OR: 5.28; 95% CI 4.45–6.26), aligning with our findings. These findings show that current cigarette, e-cigarette, and alcohol use consistently serve as predictors of high-risk sexual behavior, thereby playing a role in influencing adolescents' likelihood of engaging in sexual activity with multiple partners. One possible explanation for this is the cognitive and behavioral effects of these substances. Alcohol and nicotine are known to lower inhibitions and impair decision-making, potentially leading to impulsive behaviors, which may include engaging in sex with multiple partners (Kräplin et al., 2019). Additionally, alcohol is commonly used in party settings where social norms and peer pressure may encourage multiple sexual partnerships (Iwamoto & Smiler, 2013).

Furthermore, our findings reveal that current marijuana use is associated with a higher likelihood of having condomless sex, similar to Schumacher et al. (2018) study. Schumacher's meta-analysis of eleven U.S. studies found a statistically significant association between marijuana use and lower odds of using a condom in the overall pooled analysis (OR: 0.71; 95% CI 0.56–0.89). Stratified analyses revealed that adolescents who used marijuana were significantly less likely to use a condom (OR: 0.62; 95% CI 0.47–0.82). However, the pooled OR for adults was not statistically significant (OR: 0.92; 95% CI 0.64–1.33. These findings could result from the fact that while marijuana also impairs cognitive function, its use may be associated with a higher likelihood of not using a condom due to altered judgment and aspects of planning, and increased risk-taking behaviors (Crean et al., 2011), making users less likely to consider or prioritize condom use in the moment.

Overall, these associations could potentially be due to the disinhibiting effects of substances, their impact on decision-making, judgement, and planning, or social factors such as peer pressure, contributing to both high-risk behaviors (Andrews et al., 2020; Whitesell et al., 2013).

A significant variation was observed in the associations between current marijuana use and non-condom use during the last sexual intercourse by sexual identity, with heterosexual students showing a stronger association compared to LGBQ + students (p = 0.010). Moreover, no significant association was identified for LGBQ + students in this context, a finding consistent with Clayton et al. (2019), who similarly reported non-significant associations between current marijuana use and no condom use during last sexual intercourse among sexual minority youths. Clayton et al. (2019) also highlighted a stronger association for heterosexual students, mirroring the current study's findings. These variations underscore the complexity of the interplay between substance use and sexual behavior across diverse identity groups, indicating a need for targeted interventions among heterosexual adolescents. Findings may also reflect unique protective or risk-amplifying factors, such as access to LGBTQ + -specific health resources, differing social norms, or increased awareness of sexual health risks within these communities, especially since sexual minorities are at an increased risk for sexually transmitted infections such as HIV (Wood et al., 2016).

The observed associations between current substance use and high-risk sexual behaviors have important implications for prevention strategies targeting U.S. high school students. Given that cigarette, e-cigarette, alcohol, and marijuana use are linked with having multiple sexual partners or engaging in condomless sex, school- and community-based programs should prioritize the early identification of at-risk adolescents. Interventions that integrate substance use education with sexual health promotion may be particularly effective, as evidence suggests that combining these approaches can reduce both substance use and risky sexual behaviors among youths (Hale et al., 2014). For example, evidence-based school programs such as *Safer Choices* and *Project ALERT*, which address adolescent sexual risk behaviors and substance use, have been shown to enhance knowledge about the health consequences of these behaviors, promote prevention strategies, and reduce engagement in high-risk behaviors (Education Training Research, n.d.; Project ALERT, 2025). Additionally, community-based interventions such as The Self-Help in Eliminating Life-Threatening Diseases (SHIELD), Communities That Care, Be Proud! Be Responsible!—proven prevention programs designed to reduce adolescent substance use, risky sexual behavior, and HIV/STI infection—are equally critical. These programs have demonstrated effectiveness in randomized controlled trials when implemented by community-based organizations, showing significant reductions in risk behaviors and continued potential for broader prevention efforts (Blueprints, n.d.; Jemmott et al., 2010; The Center for Communities That Care, n.d.).

Policy-level interventions are also critical for prevention. Controversies surrounding marijuana legalization have raised concerns about its potential impact on youth risk behaviors, highlighting the need for a deeper understanding of these relationships to inform public policy. Nearly one-third (31.6%) of high school students reported current use of tobacco products, alcohol, or marijuana between January and June of 2021 (Brener et al., 2022), and approximately 80% of adolescent cannabis users reported use within the past month (Ladegard & Bhatia, 2023; Wadsworth et al., 2022). These alarming statistics reflect gaps in enforcement and suggest that existing laws fail to sufficiently protect adolescents from risky behaviors associated with substance use. Enforcing stricter age restriction laws to restrict youth access to these substances remains an urgent priority to safeguard adolescents and mitigate long-term public health consequences.

## Strengths & Limitations

This study has several strengths and limitations worth considering. Regarding strengths, the use of YRBS data provides a robust and nationally representative sample of sexually active U.S. high school students. Use of this dataset enhances the generalizability of findings to this population, and the YRBS questions used have been shown to demonstrate good test–retest reliability (Brener et al., 2002, 2013). The study also examined multiple substances (cigarettes, e-cigarettes, alcohol, and marijuana) and their associations with sexual risk behaviors, providing a multifaceted analysis of adolescent behaviors. Furthermore, the use of effect modification allowed researchers to examine variations in associations and highlighted disparities that may be specific to certain subpopulations.

Regarding limitations, the cross-sectional nature of the data prevents establishment of causality or temporal relationships between current substance use and sexual risk behaviors. Also, the data provided by respondents were self-reported and therefore could introduce social desirability bias leading to overreporting or underreporting of behaviors. Furthermore, the results are only generalizable to sexually active adolescents attending high school and do not represent all youth in the U.S. Finally, data were dichotomized into two broad race categories (White and non-White) for ease of analysis, limiting insights into specific non-White racial groups and preventing a more detailed understanding of associations among these subpopulations.

## Conclusion

Findings indicate an association between current substance use and high-risk sexual behaviors among sexually active U.S. adolescents, with no significant variation by race or sex. Compared to their LGBQ + peers, a stronger association between marijuana use and no condom use was found among heterosexual students. Tailored interventions targeting substance use prevention and sexual health education are crucial to address these interconnected health risks. Special emphasis should be placed on how use of particular substances put youth at risk for specific sexual behaviors, and certain demographic groups may require targeted programming efforts based on unique needs. Continued research is essential to explore these associations comprehensively across other stratified sociodemographic groups and over a longer period using a longitudinal study design to inform equitable public health strategies.
